# Supplementary material for: The genome sequence of the Mottled Pug, Eupithecia exiguata (Hübner, 1813)
Source: Wellcome Open Res. 2024 Feb 19;9:65. [Version 1] doi: 10.12688/wellcomeopenres.20637.1 (PMC11249503; doi:10.12688/wellcomeopenres.20637.1)
Supplement: Peer review history

# Reviewer report: Reviewer response for version 1

**DOI:** 10.21956/wellcomeopenres.22840.r82099

**Published:** 2024-07-15

**Authors:** Saskia Wutke

**Affiliations:** 1 University or Eastern Finland, Joensuu, Finland

This data note reports the high-quality genome of the geometrid moth *Eupithecia exiguata* (Mottled Pug). Genome sequencing, assembly and annotation is based PacBio-HiFi and Hi-C data. Overall, the dataset is presented clearly and concisely. The methods are state-of-the-art and follow the usual DToL procedures. Nevertheless, as with other DToL genome reports, I would wish for more details in the method section, particularly concerning the software settings and the protein set used for genome annotation. Likewise, the background could be more elaborate with some explanation about its relevance. It seems like the background section of DToL genome reports gets shorter and shorter.

 Still, the genome provides a valuable resource for evolutionary and comparative genomic research.

Are sufficient details of methods and materials provided to allow replication by others?

Partly

Is the rationale for creating the dataset(s) clearly described?

Yes

Are the datasets clearly presented in a useable and accessible format?

Yes

Are the protocols appropriate and is the work technically sound?

Yes

Reviewer Expertise:

Genome sequencing, genomics, phylogenomics, Hymenoptera

I confirm that I have read this submission and believe that I have an appropriate level of expertise to confirm that it is of an acceptable scientific standard.

# Reviewer report: Reviewer response for version 1

**DOI:** 10.21956/wellcomeopenres.22840.r78189

**Published:** 2024-05-16

**Authors:** Jaakko Pohjoismäki

**Affiliations:** 1 University of Eastern Finland, Joensuu, Finland

The manuscript by Boyes, Lewis & the DToL consortium presents the reference-level genome assembly for the geometrid moth *Eupithecia exiguata*. The genome quality and assembly corresponds to the standards of previous reference genome releases and has been obtained using the standard DToL pipeline.

 Unfortunately the genome is again from a male and thus lacks the W sex chromosome. I think that more effort should be put into obtaining the heterogametic sex for the reference genomes, as sequencing another reference genome from the same species is unlikely or at least low priority. Also this species is very common and can be also found resting on walls etc. during days, so obtaining a female is not very difficult. Otherwise I have only minor suggestions:

 The background is quite sparingly written. *Eupithecia* is a very species-rich genus, with diverse host plant associations and habitat requirements. Some more information about the diversity in the UK would be useful.  Also as some (or most) species can be difficult to determine, it would be important to explain how *exiguata *can be differentiated from other similar species. Despite these minor shortcomings, this high quality genome assembly is a nice addition to the growing number of available lepidopteran reference genomes.

Are sufficient details of methods and materials provided to allow replication by others?

Yes

Is the rationale for creating the dataset(s) clearly described?

Yes

Are the datasets clearly presented in a useable and accessible format?

Yes

Are the protocols appropriate and is the work technically sound?

Yes

Reviewer Expertise:

mitochondrial DNA, biodiversity genomics, taxonomy

I confirm that I have read this submission and believe that I have an appropriate level of expertise to confirm that it is of an acceptable scientific standard, however I have significant reservations, as outlined above.

# Reviewer report: Reviewer response for version 1

**DOI:** 10.21956/wellcomeopenres.22840.r78208

**Published:** 2024-04-11

**Authors:** Marco Gerdol

**Affiliations:** 1 University of Trieste, Trieste, Italy

This manuscript reports a high-quality genome assembly of the Mottled Pug Eupithecia exiguata, using the standardized format of the Darwin Tree of Life genome reports. This resource was obtained through the application of gold standard methodologies and the metrics of the assembly itself are excellent. Compared with other similar papers, I also appreciate the presence of a brief gene annotation summary, created with the Ensembl rapid pipeline. There are, however, a few points that would benefit from some additional clarification:

- It is unclear whether the number of pseudo chromosomal scaffolds does actually match the expected number of chromosomes from previous cytogenetic studies (if available).
- The authors mentioned that genome annotation was aided by transcriptomic data, but it is unclear whether this data was generated within the frame of this study, or it was previously available. In both cases, the materials and methods section should specify the accession IDs of RNA-seq of the datasets used for this purpose.
- The data availability section should include a link to the Ensembl rapid webpage with the genome annotation.
- It would be useful also to briefly mentioned the observed level of heterozygosity in the results section.

Are sufficient details of methods and materials provided to allow replication by others?

Yes

Is the rationale for creating the dataset(s) clearly described?

Yes

Are the datasets clearly presented in a useable and accessible format?

Partly

Are the protocols appropriate and is the work technically sound?

Yes

Reviewer Expertise:

Invertebrate genomics

I confirm that I have read this submission and believe that I have an appropriate level of expertise to confirm that it is of an acceptable scientific standard, however I have significant reservations, as outlined above.
